# Moderate Exercise Modulates Inflammatory Responses and Improves Survival in a Murine Model of Acute Pneumonia*

**DOI:** 10.1097/CCM.0000000000006166

**Published:** 2024-01-09

**Authors:** Veronique Vermeersch, Karelle Léon, Anais Caillard, Amandine Szczesnowski, Gaëlle Albacete, Nadege Marec, Florine Tissier, Guillaume Gilbert, Mickael Droguet, Pascale Marcorelles, Marie-Agnes Giroux-Metges, Olivier Huet

**Affiliations:** 1 Department of Anesthesia and Intensive Care Unit, Brest Teaching Hospital, Brest, France.; 2 ORPHY, EA4324, Université de Bretagne Occidentale, Brest, France.; 3 LBAI, Inserm UMR1227, Université de Bretagne Occidentale, Brest, France.; 4 Department of Pathology, Brest Teaching Hospital, Brest, France.; 5 Explorations Fonctionnelles Respiratoires, Brest Teaching Hospital, Brest, France.; 6 Australian and New Zealand Intensive Care research Center, Monash University, Melbourne, VIC, Australia.

**Keywords:** cytokines, innate immune response, macrophage, moderate physical exercise, pneumonia

## Abstract

**OBJECTIVES:**

An association between physical inactivity and worse outcome during infectious disease has been reported. The effect of moderate exercise preconditioning on the immune response during an acute pneumonia in a murine model was evaluated.

**SETTING:**

Laboratory experiments.

**SUBJECTS:**

C57BL6/j male mice.

**INTERVENTIONS:**

Six-week-old C57BL/6J mice were divided in two groups: an exercise group and a control group. In the exercise group, a moderate, progressive, and standardized physical exercise was applied for 8 weeks. It consisted in a daily treadmill training lasting 60 minutes and with an intensity of 65% of the maximal theoretical oxygen uptake. Usual housing recommendation were applied in the control group during the same period. After 8 weeks, pneumonia was induced in both groups by intratracheal instillation of a fixed concentration of a *Klebsiella pneumoniae* (5 × 103 colony-forming unit) solution.

**MEASUREMENTS AND MAIN RESULTS:**

Mice preconditioned by physical exercise had a less sever onset of pneumonia as shown by a significant decrease of the Mouse Clinical Assessment Severity Score and had a significantly lower mortality compared with the control group (27% vs. 83%; *p* = 0.019). In the exercise group, we observed a significantly earlier but transient recruitment of inflammatory immune cells with a significant increase of neutrophils, CD4+ cells and interstitial macrophages counts compared with control group. Lung tumor necrosis factor-α, interleukin (IL)-1β, IL-6, and IL-10 were significantly decreased at 48 hours after pneumonia induction in the exercise group compared with the control group.

**CONCLUSIONS:**

In our model, preconditioning by moderate physical exercise improves outcome by reducing the severity of acute pneumonia with an increased but transient activation of the innate immune response.

**KEY POINTS**

**Question:** Does preconditioning by moderate physical exercise influences the immune response at the early stage of infection?

**Finding:** Mice preconditioned by moderate physical exercise had a significantly lower risk of mortality after sever pneumonia induction. An earlier but transient recruitment of inflammatory immune cells including anti-inflammatory phenotyped macrophages may explain this protective effect.

**Meaning:** In a context of pandemic and increase in infectious diseases susceptibility, moderate physical exercise can be useful to prevent sever form of infection.

Despite improvement of patients care, mortality due to severe infection remains high (1, 2). Tremendous efforts have been made in fundamental and clinical research to develop novel therapeutic to decrease this burden without significant success. Prevention has become an area of increased interest. However, defining the right prevention strategy requires a better understanding of host and pathogens interactions to foster an optimal response in case of infection.

It has been reported that during community-acquired pneumonia the early and abnormal elevations of pro- and anti-inflammatory cytokines are associated with an increased risk of developing sepsis and an increased mortality (3–5).

Thus, strategy promoting an appropriate immune response could participate in the prevention of severe infection. Actually, a timely appropriate and localized immune response is a key host event to contain and eradicate the infection (6). Huet et al (7) reported in a murine model of pneumoniae that an early and transient increase of the inflammatory response is essential to activate the innate immunity and avoid the development of a lethal systemic inflammatory response.

It has been demonstrated that physical exercise can decrease postoperative infections after cardiac surgery (8). Physical exercise influences the inflammatory response and is part of the body’s adaptation to stress. Prophylactic physical exercise may protect against inflammation and oxidative stress in rats (9) and improves inflammation induced by lipopolysaccharide (LPS) by regulating Warburg effect in mice (10). It has also been reported that physical exercise decreased the production of inflammatory parameters, leading to a reduction in tissue damage and an improvement in survival in a murine model of endotoxemia (11). In a mice model, Zhang et al (12) showed that a physical preconditioning significantly attenuates liver injury and inflammation from ischemia and reperfusion. Reports have also showed that physical exercise modulates the immune response after LPS stimulation (13, 14). These finding suggests that moderate exercise may be beneficial in case of infection or acute inflammation. However, exact mechanisms of this effect need to be better understood.

In this study, we evaluated the effect of moderate physical preconditioning on the activation and modulation of the inflammatory response in a model of acute pneumonia. To answer this, the objective of this study was to identify the effect of physical activity on the lung of immune response during acute pneumonia.

## MATERIALS AND METHODS

### Animals

C57BL/6J male mice from 6 to 7 weeks old, weighting 18–24 g were used. Animals were maintained under day-night cycles (12:12 hr light-dark cycle; 23°C ± 1°C room temperature; 40–60% environment humidity) and received a standard pellet rodent diet and water ad libitum. Experimental procedures were approved by the local Animal Ethics Committee n°074, protocol number 13891-2018012211053283. Mice were killed by intraperitoneal injection of pentobarbital at indicated time points before and after infection for analysis, such as bacterial colony-forming unit (CFU) assay, flow cytometry, and cytokine analysis.

### Exercise Training

Mice were randomly assigned to two groups: control and exercise. In the exercise group intervention consisted in a regular and moderate physical exercise, at 65% of theoretical maximal oxygen uptake on a treadmill (Domyos; Decathlon Brest, Guipavas, France). After 1 week of progressive familiarization with the treadmill, the mice followed an 8 weeks running protocol according to the following model: 60 minutes of daily running, 5 days a week, at a speed of 0.5 km/hr for the first 4 weeks, and 0.8 km/hr for the following 4 weeks.

### Bacterial Growth

*Klebsiella pneumoniae* (serotype K2 [American Type Culture Collection (ATCC) 43816]) was cultured overnight in Luria-Bertani broth at 37°C. After centrifugation, the Luria-Bertani media was removed, and the bacterial pellet was washed twice (1000 g, 10 min, 4°C), diluted in sterile isotonic saline, and calibrated by spectrophotometry in 0.9% saline solution.

### Induction of Pneumonia

Induction of pneumonia has been previously described (14). Briefly, anesthesia was performed by an intraperitoneal injection of ketamine (80 mg/kg), xylazine (10 mg/kg), and atropine (0.3 mg/kg). Cervical area dissection was made to access to the trachea and *K. pneumoniae* (5 × 103 CFUs) was then injected intratracheally.

### Mortality Assessment

To assess the evolution of the infection and the mortality risk, we used a previously described scoring system: the Mouse Clinical Assessment Severity Score (14).

The scoring sheet was designed using eight humane endpoints described in the literature (15–19). A definition of each parameter is given (**Table S1**, http://links.lww.com/CCM/H474). For each parameter, four stages representing the evolution from a healthy to a severely ill mouse were defined. When mice reached a score of 4 in the eight parameters they were euthanized. If a mouse reached a score of 4 on one items at night, it was euthanized to prevent overnight death.

Two different blinded examiners performed the analysis of the severity score.

### Bacterial Concentration in Lung Homogenates

Mice (*n* = 9 per group) were euthanized, and lungs were harvested. Collected lungs were homogenized (VWR homogenizer, Radnor, PA) in 3 mL of 0.9% saline solution. Bacterial loads were determined by plating serial dilutions of total lung homogenate on Luria-Bertani agar plates. Dilutions were plated in duplicate. The dishes were incubated at 37°C for 24 hours then the CFUs were counted. Results are presented as log CFU/g of lung ± sem.

### Cytokines Measurements

After induction of pneumonia, mice (*n* = 6 per group) were euthanized. Blood collected by cardiac puncture was centrifuged (1000 revolutions/min for 10 min at 4°C), and the plasma was collected and frozen at –20°C until analysis. Pulmonary cytokines were measured in lung homogenate supernatant.

The concentrations of cytokines were measured using Millipore Milliplex map kit, mouse high sensitivity premixed panel (MHSTCMAG-70KPMX) as manufacturer’s instructions. The plate was read using BioRad BioPlex System (Luminex, Austin, TX) and the BioPlex Manager program, Version 4 (Bio-Rad, Hercules, CA).

### Histology

Lungs were harvested, formalin fixed, and paraffin embedded as previously described (20, 21). They were next stained with hematoxylin and eosin (H&E) and analyzed for inflammatory polynuclear and lymphocytes infiltrate (score from 0 to 4). Alveolus, bronchus, and parenchyma structures were identified. Lymphocytes and polynuclear cells were distinguished by morphology, and infiltration in peribronchiolar and alveolar zone were studied at a magnification of 40. Three sections from the right lung and two sections from the left lung were analyzed using a histologic severity score (**Table S2**, http://links.lww.com/CCM/H474) according to Yatmaz et al (21). Six to seven mice lungs were analyzed per group. A pathologist, blinded to the allocation groups, performed the analysis of the slides.

### Flow Cytometry

After euthanasia lungs were harvested (*n* = 6–12 per groups). Lung preparation, analytical, and preparative flow cytometry were performed as already described (22). Briefly, mice were euthanized, and lungs were harvested. Lungs were carefully sheared then digested for 45 minutes at 37°C with Roswell Park Memorial Institute-1640 (Thermo Fisher Scientific, Waltham, MA) containing collagenase (Worthington Biochemical, Lakewood, NJ) and deoxyribonuclease (Sigma-Aldrich, St. Louis, MO). The suspension was then filtered through 70 μm filters (Corning, Corning, NY). RBCs were lysed with RBC lysis solution (BioLegend, San Diego, CA) and then removed after centrifugation. Cell suspensions were washed twice in fluorescence-activated cell sorting preparation and were processed according to the manufacturer’s instructions for flow cytometry. The following conjugated monoclonal antibodies (BioLegend) were used (provided as name, clone, lot number, dilution): CD103-Alexa Fluor (AF) 700, 2E7, 121442, 1:200; CD11c-APC, N418, 117309, 1:200; CD4-allophycocyanin (APC)/Fire 750, rat monoclonal (RM) 4-4, 116019, 1:200; Ly-6G-APC/Fire750, 1A8, 127651, 1:200; CD24-brilliant violet (BV) 421, M1/69, 101826, 1:200; CD25-BV421, polychromatic 61, 102043, 1:200; CD19-BV510, 6D5, 115546, 1:200; CD3-BV510, 17A2, 100234, 1:200; CD8a-BV510, 53-6.7, 100752, 1:200; Natural Killer (NK) 1.1-BV510, protein kinase (PK) 136, 108738, 1:200; T-cell receptor β-BV605, H57-597, 109241, 1:200; CD3-BV605, 17A2, 100229, 1:200; CD45.2-fluorescein isothiocyanate, 104, 109805, 1:200, major histocompatibility complex class II, I-Ab (a specific allele) conjugated with phycoerythrin (PE), AF6-120.1, 116407, 1:200; NK1.1,-PE, PK136, 108707, 1:200, Ly-6C, HK1.4, 128017, 1:200; CD19-PE/Dazzle594, 6D5, 115553, 1:200; CD11b-PE/Dazzle594, M1/70, 101255, 1:200; and anti-mouse CD16/32, 93, 101319, 1:100.

Samples were acquired on a Cytoflex S flow cytometer (Beckman Coulter, Brea, CA). They were analyzed using Kaluza software (Version 2.1; Beckman Coulter).

### Phagocytosis Assay for Alveolar Macrophages

*Escherichia coli* green fluorescent protein (ATCC 25922GFP) (GFP-*E. coli*) were grown overnight in Tryptic Soy broth media with 100 µg/mL ampicillin.

For the in vitro phagocytosis assay, mice were euthanized and bronchoalveolar lavage was collected. Alveolar macrophages (AMs) of control or exercised mice (*n* = 6 per group) were infected with GFP-*E. coli* (multiplicity of infection of 1) for 2 hours at 37°C. Phagocytic AMs percentage (F4/80+CD11d+) was determined by flow cytometry 2 hours after the in vitro infection.

For in vivo phagocytosis assay, GFP-*E. coli* were intratracheally injected (optical density at 600 nanometers = 1. 35 µL). Lungs were next harvested and percentage of GFP macrophages were measured by flow cytometry as previously described (23). The bacterial load was verified by plating ten-fold serial dilutions on Tryptic Soy broth agar plates.

### Statistical Analysis

Differences in survival were assessed by log-rank analysis and represented by Kaplan-Meier curves. All analyses were performed by using GraphPad Prism (Version 9; GraphPad Software, La Jolla, CA). Statistical parameters including the definition of center, dispersion and precision measures, and statistical significance are reported in figures. Unpaired *t* test and unpaired Mann-Whitney *U* test with two-tailed *p* values and 95% CIs were performed to compare groups. One-way analysis of variance with Tukey test was performed to compare more than two groups. A *p* value of less than 0.05 was considered significant (*p < 0.05, **p < 0.01, ***p < 0.001, ****p < 0.0001). Error bars represent sem.

## RESULTS

**Figure 1.**
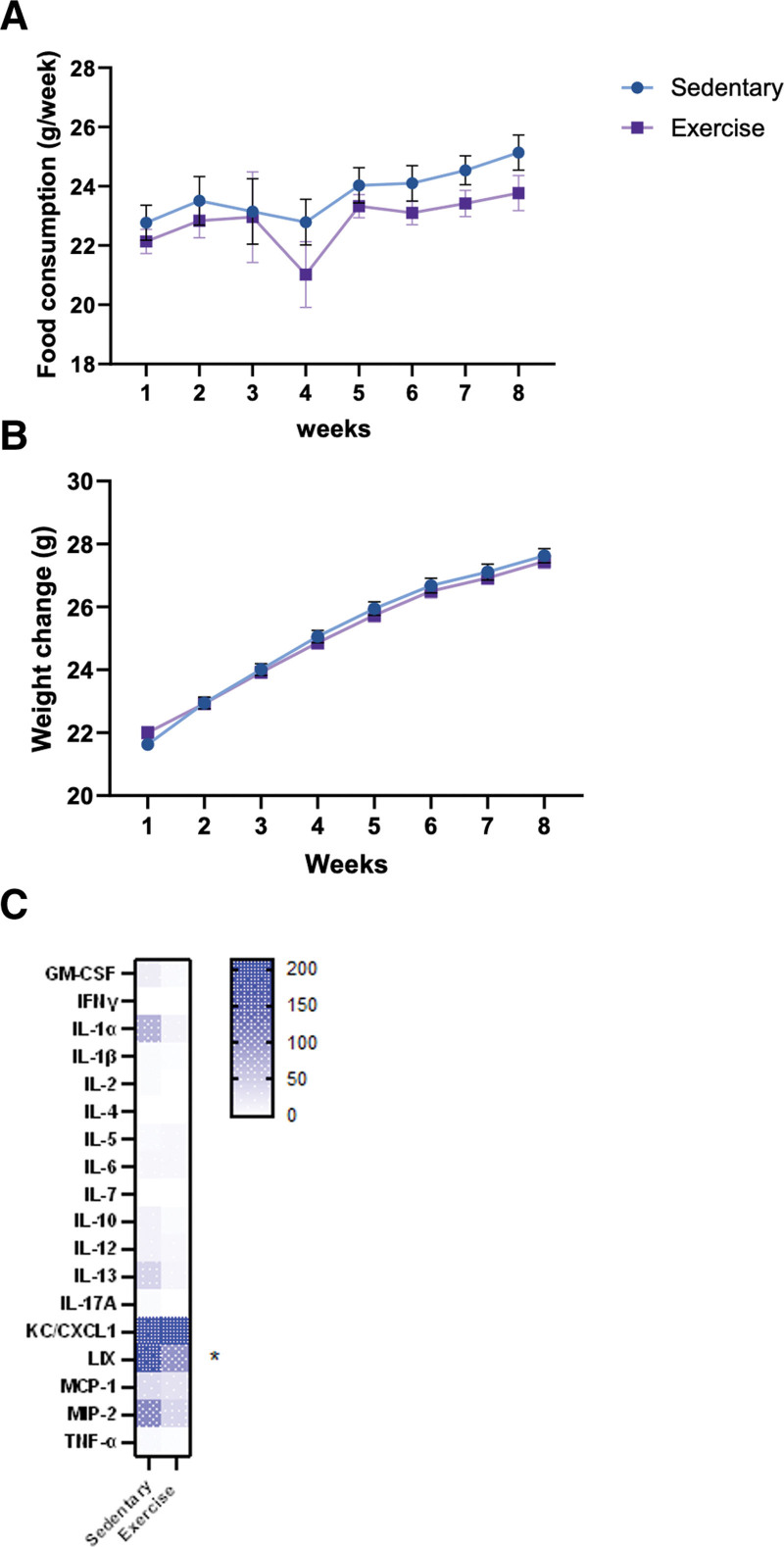
Metabolism and inflammation at basal state. **A**, Food consumption during 8 wk of exercise (*n* = 56 mice per group). **B**, Weight variation during 8 wk of exercise (*n* = 56 mice per group). **C**, Heatmap of plasma circulating cytokines after exercise period or sedentary period. (*n* = 5–6 per group). *p < 0.05. GM-CSF = granulocyte-macrophage colony-stimulating factor, IFNγ = interferon gamma, IL = interleukin, KC/CXCL1 = keratinocyte-derived chemokine (KC) (also known as CXCL1 [chemokine C-X-C motif ligand 1]), LIX = lipopolysaccharide-induced CXC chemokine, MCP-1 = monocyte chemoattractant protein-1, MP-2 = macrophage inflammatory protein-2, TNF-α = tumor necrosis factor alpha.

Exercise preconditioning using a motorized treadmill did not affect baseline characteristics of the mice compared with control group. Moderate physical exercise is well tolerated and does not modify body weight (**Fig. 1*A***) or food consumption (**Fig. 1*B***). Plasma cytokines profiling did not show difference between control and exercised mice except for the murine neutrophil-chemoattractant chemokines lipopolysaccharide-induced CXC chemokine (*p* < 0.05; **Fig. 1*C***).

**Figure 2.**
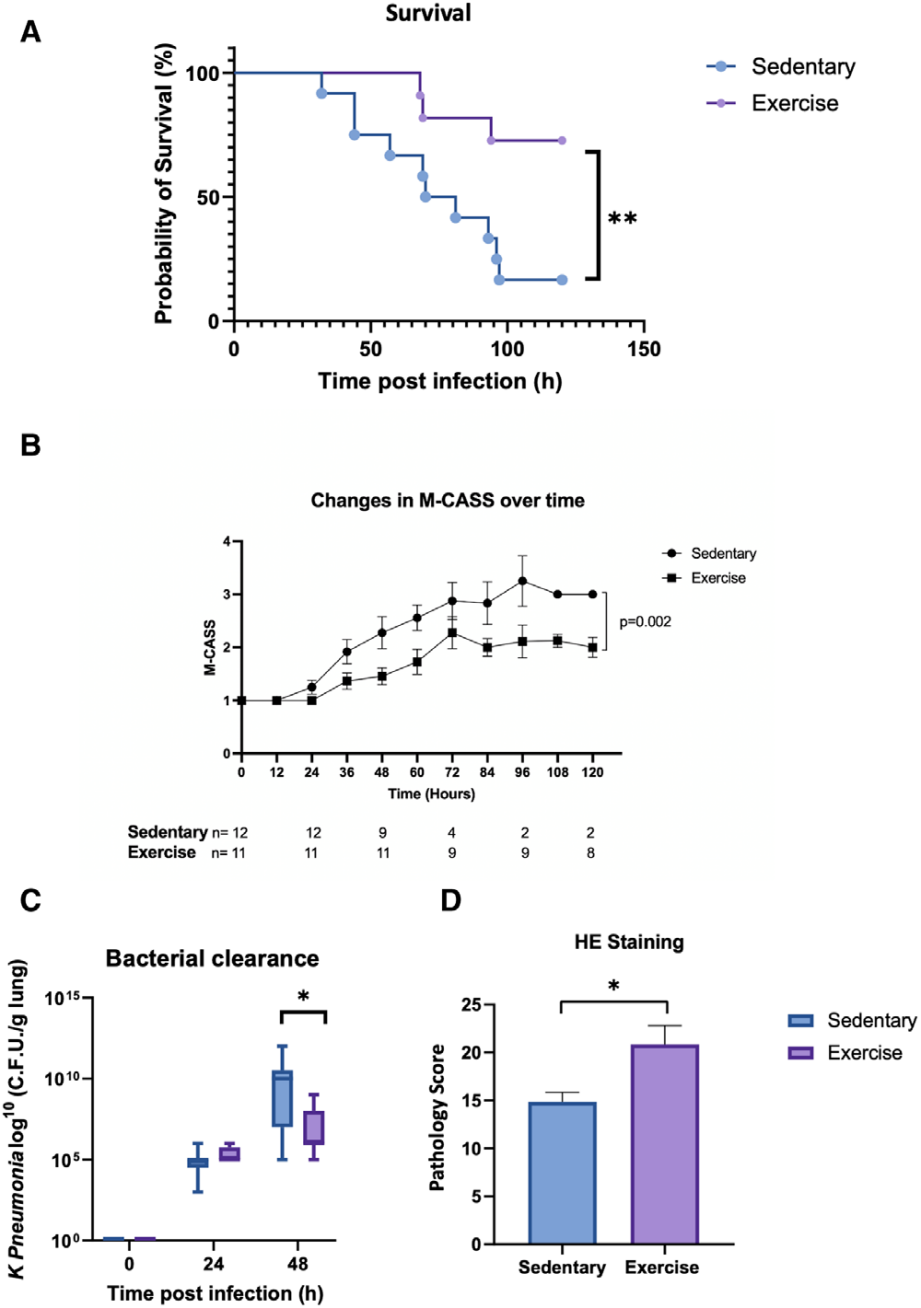
The exercise modifies the prognosis associated with sepsis. **A**, Survival after pneumonia induction (*n* = 12 sedentary and *n* = 11 exercise mice). **p < 0.01. **B**, Mouse Clinical Assessment Severity Score (M-CASS) between sedentary and exercise group (*n* = 12 or 11 per group). **C**, Number of colony-forming unit (CFU) per g of lung during pneumonia in sedentary or exercise mice (*n* = 9 per group at 24 and 48 hr). *p < 0.05. **D**, Histological analysis using hematoxylin and eosin (HE) of *Klebsiella pneumoniae*–infected sedentary and exercise mice lungs (*n* = 6 per group). *p < 0.05.

**Figure 3.**
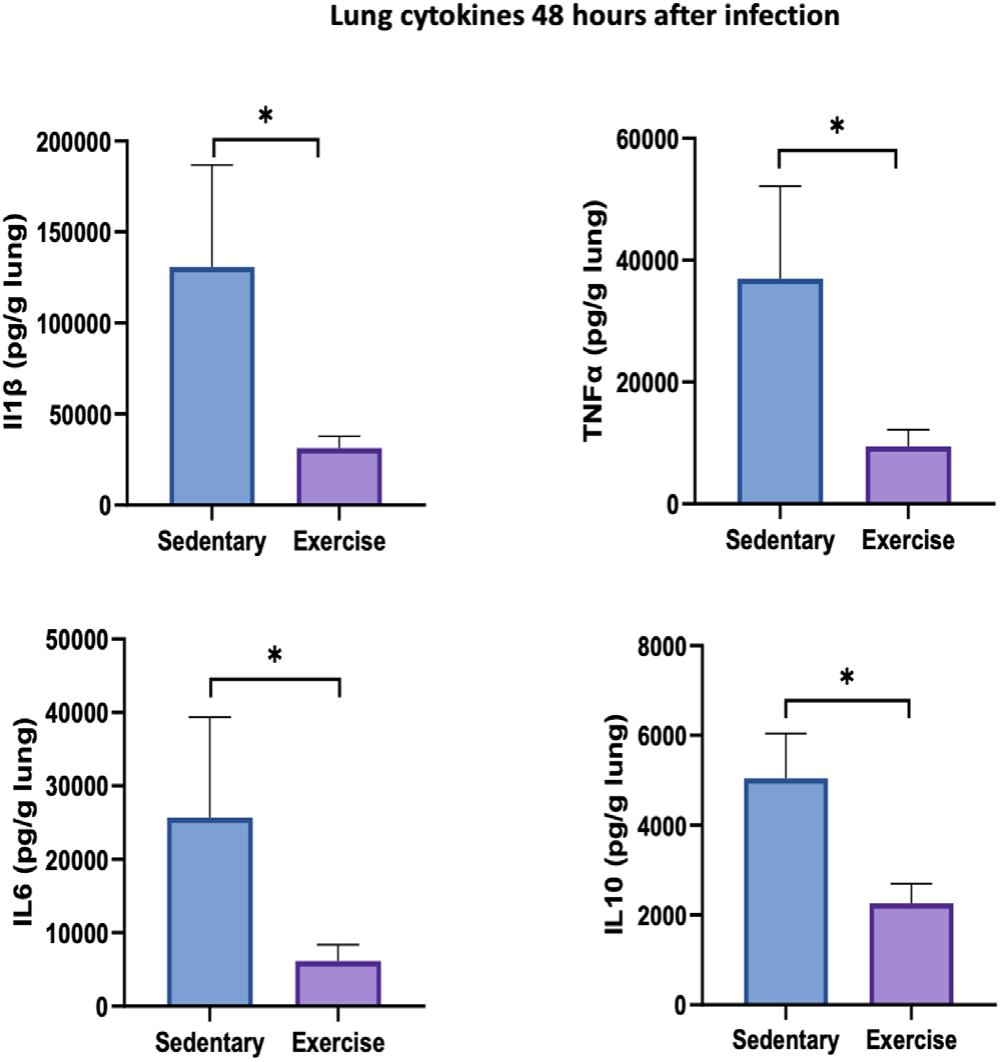
Lung cytokines 48 hr after infection. Cytokines concentration per g of lung 48 hr after pneumonia induction in sedentary and exercise group. *p < 0.05 (*n* = 9 per group). IL = interleukin, TNF-α = tumor necrosis factor alpha.

Mortality due to acute pneumonia was significantly lower in moderate exercised mice compared with control mice (27% vs. 83%; *p* = 0.019; **Fig. 2*A***). Mortality decrease was associated with a lower clinical severity score in moderate exercised mice compare to control mice (mixed model; *p* = 0.002; **Fig. 2*B***). Lung bacterial clearance was significantly increased at 48 hours in the exercised group compared with control (6.8 ± 0.4 vs. 8.89 ± 0.75 CFU/g of lung; *p* = 0.035; **Fig. 2*C***). Inflammatory infiltrate assessed by H&E staining was significantly lower in the exercised mice compared with control group (14 ± 1 vs. 20 ± 1.9; *p* = 0.02; **Fig. 2*D***). Bacterial load in the spleen 24 hours after infection was similar in the two groups (**Fig. S1**, http://links.lww.com/CCM/H474). Cytokine and chemokine dosages in lung homogenate were significantly lower at 48 hours after pneumonia induction, in the exercised group compared with control (interleukin [IL]-1β: 31.2 ± 65.4 vs. 13.1 ± 56.1 µg/g of lung, *p* = 0.04; tumor necrosis factor-α: 37 ± 15.14 vs. 94.35 ± 27.5 µg/g of lung, *p* = 0.046; IL-6: 6.2 ± 2.2 vs. 25.7 ± 13.7 µg/g of lung, *p* = 0.047; and IL-10: 2.254 ± 0.445 vs. 5 ± 1 µg/g of lung, *p* = 0.02; **Fig. 3**).

Live lung immune cell population was identified by expression marker CD45 and separated from other lung cells populations. AMs, interstitial macrophages (IMs), neutrophils, B cells, CD4+ T cells, CD8+ T cells, and natural killer cells were identified in the CD45+ lung immune cell population from both exercised and control mice after serial gating strategy (**Figs. S2** and **S3**, http://links.lww.com/CCM/H474). Phenotype of AMs and IMs are represented in the **Figures S4** and **S5** (http://links.lww.com/CCM/H474). Innate and adaptive cell populations were equally represented in control and exercised mice at baseline (**Fig. S6*A***, http://links.lww.com/CCM/H474). Moderate exercise led to a significant increase of IMs 12 hours after infection compared with control in the lung (31 × 104 ± 3.6 × 104 vs. 13.1 × 104 ± 3.5 × 104 cells; *p* = 0.005; **Fig. S6*B***, http://links.lww.com/CCM/H474). At day one after infection, a significant increase in lung neutrophils (18.3 × 104 ± 20 × 104 vs. 12.5 × 104 ± 13.8 × 104 cells; *p* = 0.03) and CD 4+ T cells (9.1 × 104 ± 1.1 × 104 vs. 4.9 × 104 ± 9.6 × 103; *p* = 0.012) were observed in the exercise mice compared with control (**Fig. S6C**, http://links.lww.com/CCM/H474). On the other hand, neutrophils (10.6 × 105 ± 1.4 × 105 vs. 18.6 × 105 ± 3.7105; *p* = 0.04) and CD4+ T cells (4.4 × 104 ± 5.7 × 103 vs. 7.4 × 104 ± 1.4 × 104; *p* = 0.04) counts were significantly lower in the exercise group compared with control at day 2 after infection (**Fig. S6*D***, http://links.lww.com/CCM/H474).

**Figure 4.**
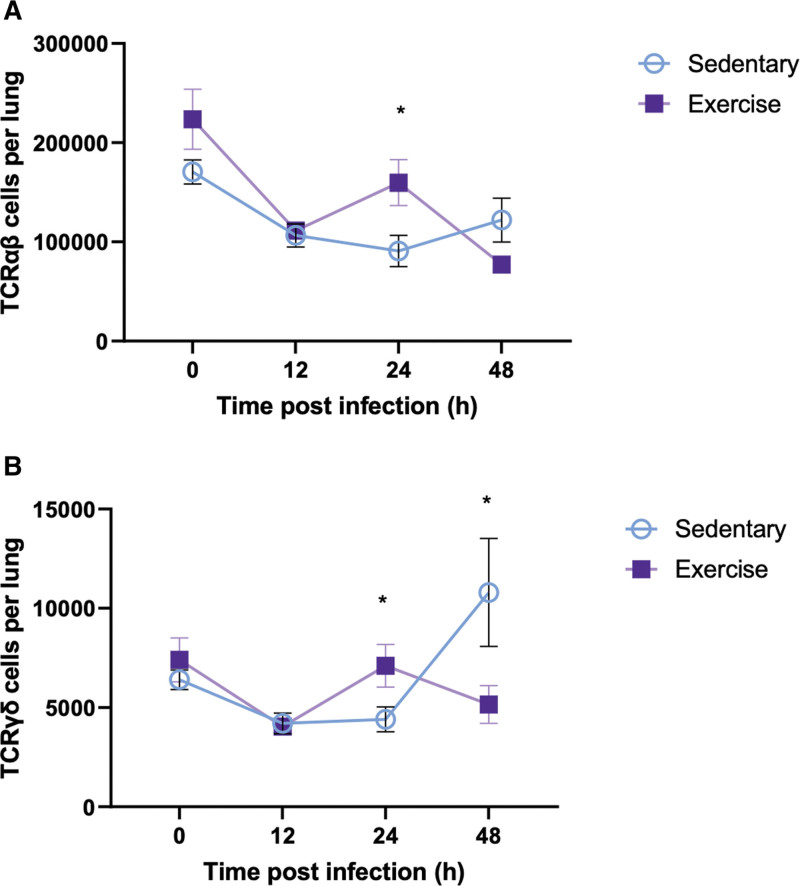
T-cell receptor evolution after infection. **A**, Absolute number of αβ T-cell receptor (TCRαβ) cells per lung at indicated time point after pneumonia induction (unpaired t test *n* = 6–12 per group). *p < 0.05. **B**, Absolute number of γδ T-cell receptor (TCRγδ) cells per lung at indicated time point after pneumonia induction (unpaired *t* test *n* = 6–12 per group). *p < 0.05.

Total number of αβ T-cell receptor (TCRαβ) and γδ T-cell receptor (TCRγδ) cells in lung tissue have been studied. Control mice and exercised mice had the same total number TCRαβ and TCRγδ cells in lung tissue at baseline. Twenty-four hours after infection induction, we observed an increase of TCRαβ cells in lung tissue in the exercise group compared with control (16 × 104 ± 2.3 × 104 vs. 9.1 × 104 ± 1.6 × 104; *p* = 0.03; **Fig. 4*A***). TCRγδ was significantly increased at 24 hours in the exercise group (7.1 × 103 ± 1.1 × 103 vs. 4.4 × 103 ± 6.2 × 103; *p* = 0.04) and significantly decreased at 48 hours (5.2 × 103 ± 952 vs. 10.9 × 103 ± 2.7 × 103; *p* = 0.02) compared with control (**Fig. 4*B***).

**Figure 5.**
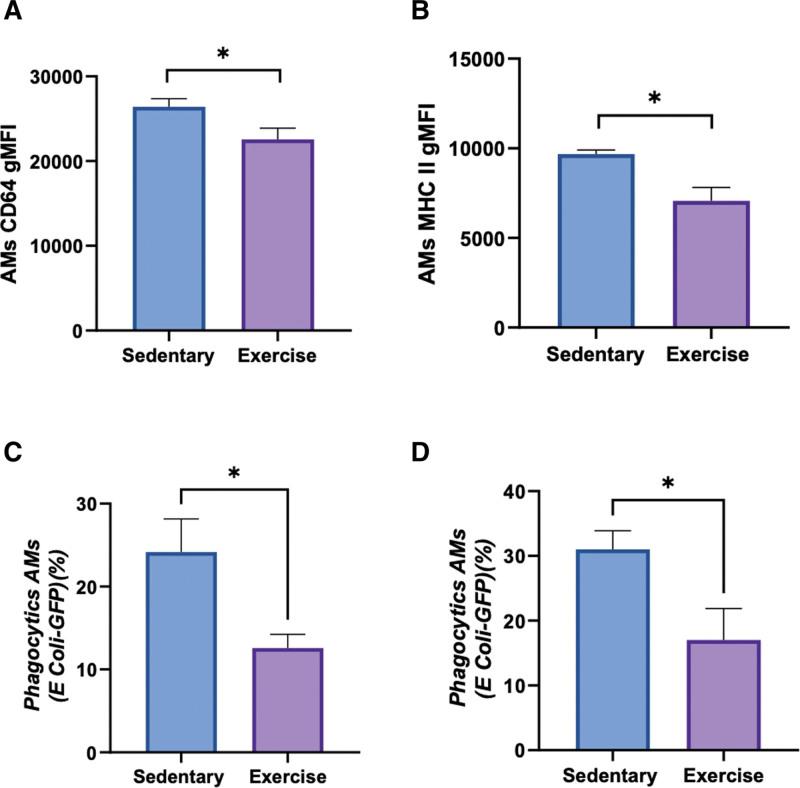
Alveolar macrophages (AMs) phenotype. **A**, CD64 expression on AMs, in the lungs of sedentary and exercise mice (*n* = 6 mice per group). *p < 0.05. **B**, Major histocompatibility complex (MHC) II expression on AMs, in the lungs of sedentary and exercise mice (*n* = 6 mice per group). *p < 0.05. **C**, Percentages of phagocytic AMs 2 hr after in vitro phagocytic assay of cells positive for green fluorescent protein (GFP) (GFPpos)-*Escherichia coli* with bronchoalveolar lavage of sedentary and exercised mice (*n* = 5–6 mice per groups). *p < 0.05. **D**, Percentages of phagocytic AMs after in vivo phagocytic assay of GFPpos-*E. coli* of sedentary and exercised mice (*n* = 5–6 mice per groups). *p < 0.05. gMFI = geometric mean fluorescence intensity.

To determine the impact of physical exercise on macrophage functional phenotype we studied surface markers expression. We observed a shift in the expression levels of surface markers CD64 (22.6 × 103 ± 1.3 × 103 vs. 26.4 × 103 ± 0.9 × 103; *p* = 0.04; **Fig. 5*A***) and major histocompatibility complex (MHC) class II on AMs (7.1 × 103 ± 7.5 × 103 vs. 9.7 × 103 ± 0.2 × 103; *p* = 0.02; **Fig. 5*B***) in the exercise group in comparison to the control group. When we studied phagocytic AM function in ex vivo (**Fig. 5*C***) and in in vivo conditions (**Fig. 5*D***), we observed a decreased phagocytic activity in AMs of the exercise group compared with control.

## DISCUSSION

In our study, we report that moderate exercise training improves outcome in a model of sever pneumonia. Exercise improves survival on day 5 after infection, increases lung bacterial clearance and reduces inflammatory response at 48 hours after infection. Moderate exercise mice showed an earlier recruitment of lung immune cells-like IMs, neutrophils, and CD4+ T cells.

Our study is in line with previous reports suggesting the benefits of physical activity in preventing and modulating the inflammatory response (24–26). However, these results are often found in models that may not be clinically relevant. Our experimental model closely mimics the clinical severity of pneumonia, including the 5-day mortality rate, as well as a similar balance between pro-inflammatory and anti-inflammatory responses observed in patients (3–5). This close resemblance to the clinical presentation of pneumonia strengthens the relevance of our study.

A first mechanism that can explain the protective effect observed in our model is an early lung recruitment of immune cells. This recruitment may allow an earlier and more efficient defense against pathogens (27, 28). This effect may be like the one observed with angiotensin-II through the angiotensin-II type 1 receptor pathway (29). As exercise has an influence on the expression of angiotensin-II and angiotensin-II type 1 receptor (30) this may explain the effect observed in our study.

Another mechanism possible is the immunoregulatory effect of regulatory T cells (Treg) (γδT and αβT cells). Treg cells are involved during infection and have regulatory function by interacting with other immune cells. They play a protective role in coordinating the host response during and after lung infection (31–33). Treg cells also have a central role to control neutrophils recruitment and persistent inflammation (34). This suggests a different regulation of pro-inflammatory infiltration in exercised mice at the early phase of infection.

Recently, a prolonged alteration of macrophages function has been reported after an inflammatory insult (23) and defined as “trained immunity.” It has been reported that exercise induces “anti-inflammatory trained immunity” (12) by upregulating itaconate metabolism in resident hepatic macrophages and Kupffer cells. After respiratory viral infection, the training of AMs induces an increase of the membrane expression of MHC-II (35). Our data are not in line with these results as we report that the AMs MHC-II expression was decreased in exercised mice, and this was associated with a decreased phagocytosis function. However, it is important to note that tolerance mechanism may vary depending on the experimental model and pathology as it has been reported that MHC class II-deficient mice had a better survival after infection (36). This result needs further exploration to be confirmed.

Our study has limitations. First, although the pneumonia model is well-described, it only studies infection induced by a single pathogen and in a specific location. Additionally, while the mice belong to the same strain and exhibit genetic homogeneity, heterogeneity in the immune response is observed. Therefore, translating these results to clinical setting needs to be considered cautiously as infection is often polymicrobial and not only located in the lung. Human susceptibility to infection is also extremely variable.

Furthermore, the study did not investigate the effect of antibiotics combined with exercise on bacterial clearance, which would be administered in clinical practice. As we demonstrate associations without highlighting explicit mechanisms more studies are required to better understand the underlying mechanisms exercise protective effect during infection. We did not measure cardiac function and plasma lactate levels, which would have allowed us to objectively assess the effect of exercise on the potential development of septic shock.

Physical exercise has been shown to enhance immune function and improve tissue oxygenation, which are all crucial factors in infectious disease. Implementing exercise protocols tailored to the individual patient’s capabilities could help avoid progression of infection and improve patient outcomes. It may give more time for diagnosis and therapeutic intervention before patient’s clinical state deteriorate. By bridging the gap between preclinical evidence and clinical practice, the integration of physical exercise interventions in patient management may provide a promising approach to improve patient outcomes in critical care.

Our study does not allow us to demonstrate if one of these mechanisms is dominating the others or if their association provides the protective effect observed. The mechanisms underlying the improved survival after pneumonia, in preconditioning exercised mice, are probably complex. However, we can make the hypothesis that the protective effect observe may occur during two distinct phases: first, a rapid recruitment of immune effectors leading to a transient pro-inflammatory response which controls the pathogens growth. Second, the existence of a macrophage phenotype modulation and Treg create an anti-inflammatory environment favoring early healing.

## CONCLUSIONS

In our study, we report that moderate exercise training improves survival during acute pneumonia. This protective effect was associated to a better recruitment of inflammatory cells and an anti-inflammatory environment via AMs phenotype shift. Understanding the pathophysiological mechanisms related to the modulation of the immune response after moderate exercise may be useful as it could be used as a preventive strategy in patients at risk of infection, such as chronic obstructive pulmonary disease, diabetes, or postoperative patients. In a context of world-wide increase of infectious burden, we believe that our results are of interest, however, more experimental data are required to confirm and better understand the protective effect observed in our model.

## ACKNOWLEDGMENTS

We thank Rozenn Le Berre, Geneviève Héry-Arnaud, Stéphanie Gouriou, Charles-Antoine Guilloux, Pierre Pochard, and animal care personnel for technical support. We express our gratitude to Decathlon for their valuable assistance in designing the treadmill. We extend our gratitude to Antoine Roquilly and Cédric Jacqueline from EA3826 “Host Pathogen Interactions, Inflammation, and Mucosal Immunity” for their valuable assistance and support.

## Supplementary Material

## References

[1] Paoli CJ Reynolds MA Sinha M: Epidemiology and costs of sepsis in the United States-an analysis based on timing of diagnosis and severity level. Crit Care Med. 2018; 46:1889–1897 30048332 10.1097/CCM.0000000000003342 PMC6250243

[2] Rudd KE Johnson SC Agesa KM: Global, regional, and national sepsis incidence and mortality, 1990-2017: Analysis for the Global Burden of Disease Study. Lancet. 2020; 395:200–211 31954465 10.1016/S0140-6736(19)32989-7 PMC6970225

[3] Kellum JA Kong L Fink MP; GenIMS Investigators: Understanding the inflammatory cytokine response in pneumonia and sepsis: Results of the Genetic and Inflammatory Markers of Sepsis (GenIMS) study. Arch Intern Med. 2007; 167:1655–1663 17698689 10.1001/archinte.167.15.1655 PMC4495652

[4] Zobel K Martus P Pletz MW; CAPNETZ study group: Interleukin 6, lipopolysaccharide-binding protein and interleukin 10 in the prediction of risk and etiologic patterns in patients with community-acquired pneumonia: Results from the German competence network CAPNETZ. BMC Pulm Med. 2012; 12:6 22348735 10.1186/1471-2466-12-6 PMC3311562

[5] van Dissel JT van Langevelde P Westendorp RG: Anti-inflammatory cytokine profile and mortality in febrile patients. Lancet. 1998; 351:950–953 9734942 10.1016/S0140-6736(05)60606-X

[6] Esper AM Moss M Lewis CA: The role of infection and comorbidity: Factors that influence disparities in sepsis. Crit Care Med. 2006; 34:2576–2582 16915108 10.1097/01.CCM.0000239114.50519.0E PMC3926300

[7] Huet O Pickering RJ Tikellis C: Protective effect of inflammasome activation by hydrogen peroxide in a mouse model of septic shock. Crit Care Med. 2017; 45:e184–e194 27749344 10.1097/CCM.0000000000002070

[8] Hulzebos EH Helders PJ Favie NJ: Preoperative intensive inspiratory muscle training to prevent postoperative pulmonary complications in high-risk patients undergoing CABG surgery: A randomized clinical trial. JAMA. 2006; 296:1851–1857 17047215 10.1001/jama.296.15.1851

[9] Giustina AD Rodrigues JF Bagio E: Lung-brain crosstalk in sepsis: Protective effect of prophylactic physical exercise against inflammation and oxidative stress in rats. Mol Neurobiol. 2022; 59:3860–3872 35426063 10.1007/s12035-022-02823-5

[10] Wang X Wang Z Tang D: Aerobic exercise improves LPS-induced sepsis via regulating the Warburg effect in mice. Sci Rep. 2021; 11:17772 34493741 10.1038/s41598-021-97101-0 PMC8423727

[11] Criswell DS Henry KM DiMarco NM: Chronic exercise and the pro-inflammatory response to endotoxin in the serum and heart. Immunol Lett. 2004; 95:213–220 15388263 10.1016/j.imlet.2004.07.012

[12] Zhang H Chen T Ren J: Pre-operative exercise therapy triggers anti-inflammatory trained immunity of Kupffer cells through metabolic reprogramming. Nat Metab. 2021; 3:843–858 34127858 10.1038/s42255-021-00402-x PMC8462058

[13] Antunes BM Rosa-Neto JC Batatinha HAP: Physical fitness status modulates the inflammatory proteins in peripheral blood and circulating monocytes: Role of PPAR-gamma. Sci Rep. 2020; 10:14094 32839476 10.1038/s41598-020-70731-6 PMC7445279

[14] Miron VV Bottari NB Assmann CE: Physical exercise prevents alterations in purinergic system and oxidative status in lipopolysaccharide-induced sepsis in rats. J Cell Biochem. 2019; 120:3232–3242 30230598 10.1002/jcb.27590

[15] UCI Office of Research Administration: IACUC Policy of Establishing Humane Endpoints. 2012. Available at: http://www.research.uci.edu/ora/acup/humaneEndpoints.htm

[16] ILAR: Recognizing Pain in Animals. 2012. Available at: http://dels.nas.edu/animal_pain/index.shtml

[17] Organization for Economic Co-operation and Development: Annexes 4: Clinical Signs and Conditions of Animals Requiring Action by Animal Care Staff and Study Directors. In: Guidance Documentation of the Recognition, Assessment, and Use of Clinical Signs As Humane End-Points for Experimental Animals Used in Safety Evaluation. Directorate E (Ed). Paris, France, OECD Head of publication service, 2000, p 39

[18] Nemzek JA Hugunin KM Opp MR: Modeling sepsis in the laboratory: Merging sound science with animal well-being. Comp Med. 2008; 58:120–128 18524169 PMC2703167

[19] Morton DB Griffiths PH: Guidelines on the recognition of pain, distress and discomfort in experimental animals and an hypothesis for assessment. Vet Rec. 1985; 116:431–436 3923690 10.1136/vr.116.16.431

[20] Huet O Ramsey D Miljavec S: Ensuring animal welfare while meeting scientific aims using a murine pneumonia model of septic shock. Shock. 2013; 39:488–494 23603767 10.1097/SHK.0b013e3182939831

[21] Yatmaz S Seow HJ Gualano RC: Glutathione peroxidase-1 reduces influenza A virus-induced lung inflammation. Am J Respir Cell Mol Biol. 2013; 48:17–26 23002098 10.1165/rcmb.2011-0345OC

[22] Roquilly A McWilliam HEG Jacqueline C: Local modulation of antigen-presenting cell development after resolution of pneumonia induces long-term susceptibility to secondary infections. Immunity. 2017; 47:135–147.e5 28723546 10.1016/j.immuni.2017.06.021

[23] Roquilly A Jacqueline C Davieau M: Alveolar macrophages are epigenetically altered after inflammation, leading to long-term lung immunoparalysis. Nat Immunol. 2020; 21:636–648 32424365 10.1038/s41590-020-0673-x

[24] Natale VM Brenner IK Moldoveanu AI: Effects of three different types of exercise on blood leukocyte count during and following exercise. Sao Paulo Med J. 2003; 121:9–14 12751337 10.1590/S1516-31802003000100003 PMC11108609

[25] Shephard RJ: Cytokine responses to physical activity, with particular reference to IL-6: Sources, actions, and clinical implications. Crit Rev Immunol. 2002; 22:165–182 12498381

[26] Moldoveanu AI Shephard RJ Shek PN: The cytokine response to physical activity and training. Sports Med. 2001; 31:115–144 11227979 10.2165/00007256-200131020-00004 PMC7101891

[27] Malka R Wolach B Gavrieli R: Evidence for bistable bacteria-neutrophil interaction and its clinical implications. J Clin Invest. 2012; 122:3002–3011 22820292 10.1172/JCI59832 PMC3408731

[28] Buscher K Wang H Zhang X: Protection from septic peritonitis by rapid neutrophil recruitment through omental high endothelial venules. Nat Commun. 2016; 7:10828 26940548 10.1038/ncomms10828 PMC4785224

[29] Leisman DE Privratsky JR Lehman JR: Angiotensin II enhances bacterial clearance via myeloid signaling in a murine sepsis model. Proc Natl Acad Sci U S A. 2022; 119:e2211370119 35969740 10.1073/pnas.2211370119 PMC9407661

[30] Li X Wang K: Effects of moderate‑intensity endurance exercise on angiotensin II and angiotensin II type I receptors in the rat heart. Mol Med Rep. 2017; 16:2439–2444 28656283 10.3892/mmr.2017.6864 PMC5548009

[31] Jensen IJ Sjaastad FV Griffith TS: Sepsis-induced T cell immunoparalysis: The ins and outs of impaired T cell immunity. J Immunol. 2018; 200:1543–1553 29463691 10.4049/jimmunol.1701618 PMC5826615

[32] Kirby AC Newton DJ Carding SR: Pulmonary dendritic cells and alveolar macrophages are regulated by gammadelta T cells during the resolution of S. pneumoniae-induced inflammation. J Pathol. 2007; 212:29–37 17370296 10.1002/path.2149 PMC2970901

[33] Omar T Ziltener P Chamberlain E: Mice lacking gammadelta T cells exhibit impaired clearance of Pseudomonas aeruginosa lung infection and excessive production of inflammatory cytokines. Infect Immun. 2020; 88:e00171–e00120 32229615 10.1128/IAI.00171-20 PMC7240087

[34] Venet F Chung CS Huang X: Lymphocytes in the development of lung inflammation: A role for regulatory CD4+ T cells in indirect pulmonary lung injury. J Immunol. 2009; 183:3472–3480 19641139 10.4049/jimmunol.0804119 PMC2788796

[35] Yao Y Jeyanathan M Haddadi S: Induction of autonomous memory alveolar macrophages requires T cell help and is critical to trained immunity. Cell. 2018; 175:1634–1650.e1617 30433869 10.1016/j.cell.2018.09.042

[36] LeMessurier K Hacker H Tuomanen E: Inhibition of T cells provides protection against early invasive pneumococcal disease. Infect Immun. 2010; 78:5287–5294 20855509 10.1128/IAI.00431-10 PMC2981332

