# Supplementary material for: Moderate Exercise Modulates Inflammatory Responses and Improves Survival in a Murine Model of Acute Pneumonia*
Source: Crit Care Med. 2024 Jan 9;52(3):e142–51. doi: 10.1097/CCM.0000000000006166 (PMC10876171; doi:10.1097/CCM.0000000000006166)
Supplement: Supplementary file 1 [file ccm-52-e142-s001.docx]

Table of content

[Figure S1 : Bacterial load in the spleen 24 hours after infection 2](#_Toc147387474)

[Figure S2 : Gating strategy used to identify myeloid cell in mouse lung. 3](#_Toc147387475)

[Figure S3 : Gating strategy used to identify lymphoid cell in mouse lung. 4](#_Toc147387476)

[Figure S4 : Phenotype of AMs in sedentary and exercised mice 5](#_Toc147387477)

[Figure S5 : Phenotype of IMs in sedentary and exercised mice 6](#_Toc147387478)

[Figure S6: Numbers of Neutrophils, Ams, IMs, CD4+ T cells, CD8+ T cells, B cells and NK cells at baseline and 12, 24, 48 hours after sepsis induction 7](#_Toc147387479)

[Table S1: M-CASS scoring system 8](#_Toc147387480)

[Table S2: Histologic severity score 9](#_Toc147387481)

[Table S3: Number per experiments and reasons for excluded values 9](#_Toc147387482)

# Figure S1 : Bacterial load in the spleen 24 hours after infection

#

# Figure S2 : Gating strategy used to identify myeloid cell in mouse lung.

A sequential gating strategy was used to identify populations: first dead cells was excluded, then CD45+ cells was selectioned, doublets and lineage (CD3, NK, CD19) were excluded. Neutrophils (Ly6C), alveolar marcrophage (F4/80+, CD24-, CD11c+, CD11b-) and interstitial macrophages (F4/80+, CD24-, CD11c-, CD11b+) were identified base on the expression of specific markers.


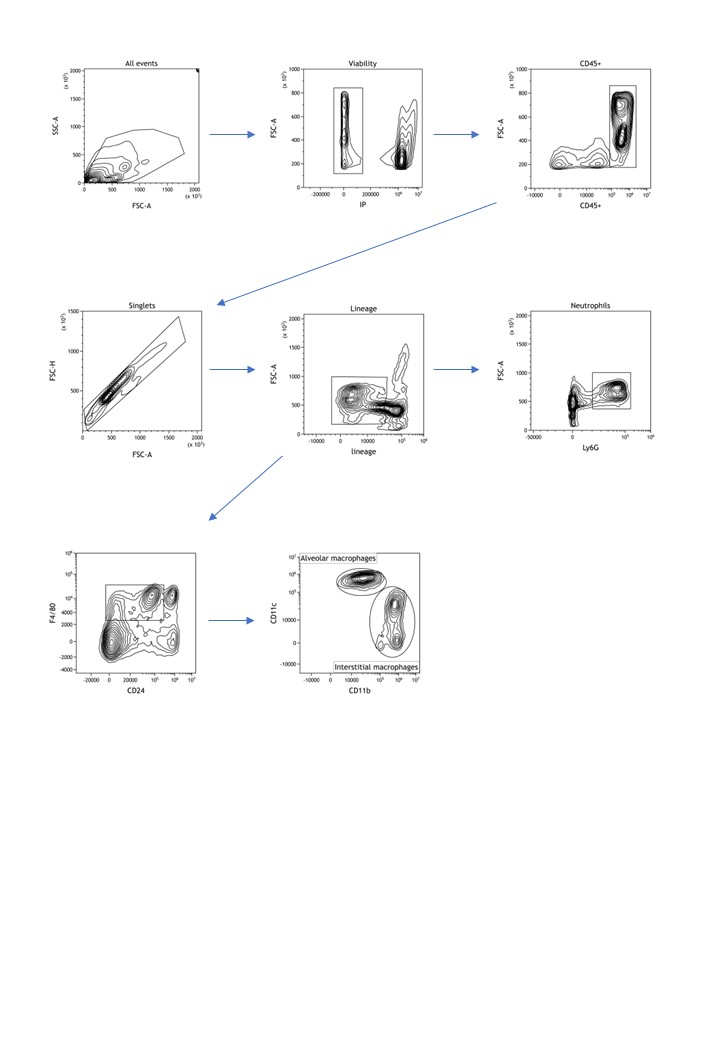


# Figure S3 : Gating strategy used to identify lymphoid cell in mouse lung.

A sequential gating strategy was used to identify populations: first dead cells was excluded, then CD45+ cells was selectioned, doublets was excluded. Natural Killer cells (NK1.1), B cells (CD19), CD4+ T Cells (CD3+CD4+), CD8+ T cells (CD3+CD8+), TCRαβ (CD3+, TCR αβ) and TCRµδ (CD3+, TCRµδ).


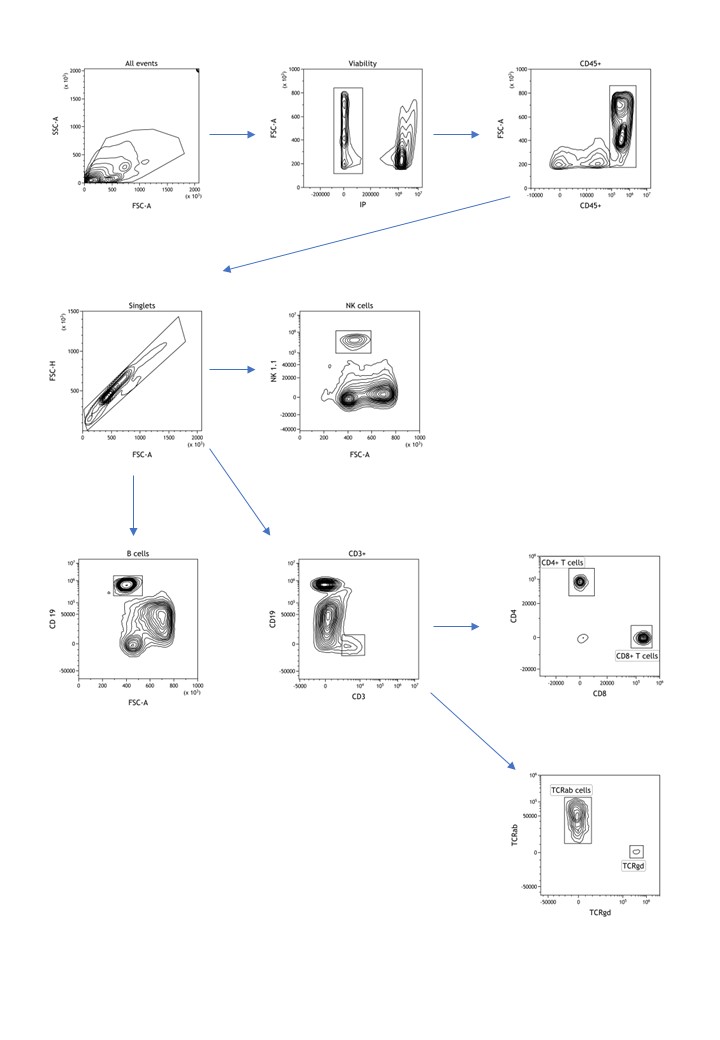


#

# Figure S4 : Phenotype of AMs in sedentary and exercised mice

CD 86, CD206, CD38 and Egr2 expressions on AMs of sedentary or exercised mice (n=6 per group). Graphs represent mean ± SEM and are pooled data from 3 independent experiments.


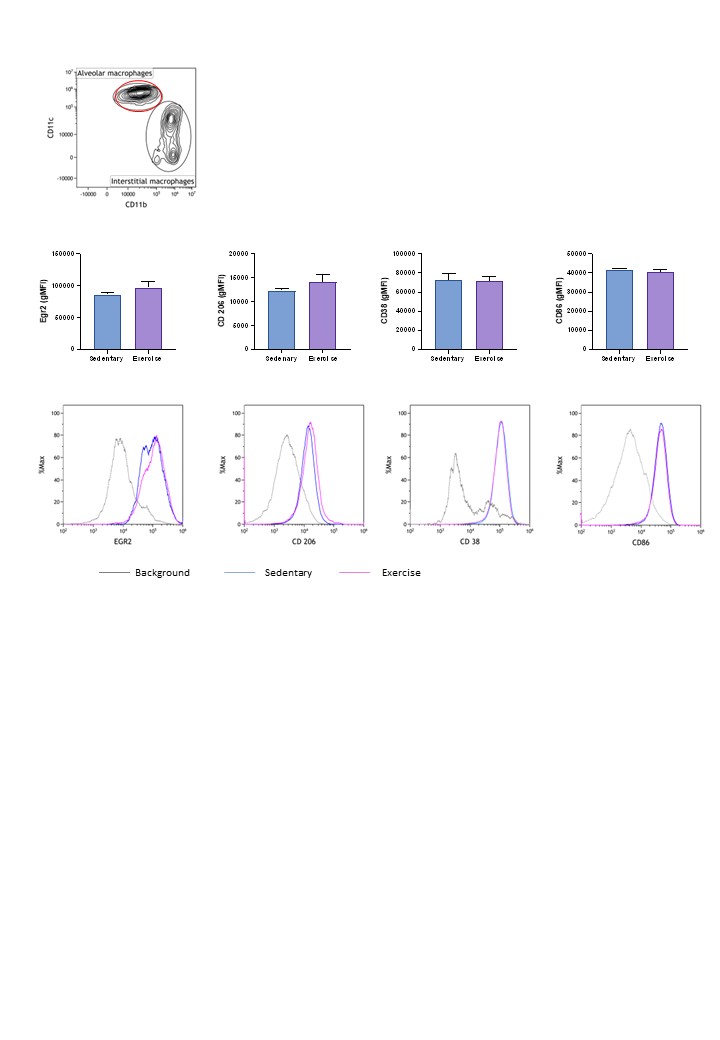


#

# Figure S5 : Phenotype of IMs in sedentary and exercised mice

CD 86, CD206, CD38 and Egr2 expressions on IMs of sedentary or exercised mice (n=6 per group). Graphs represent mean ± SEM and are pooled data from 3 independent experiments.


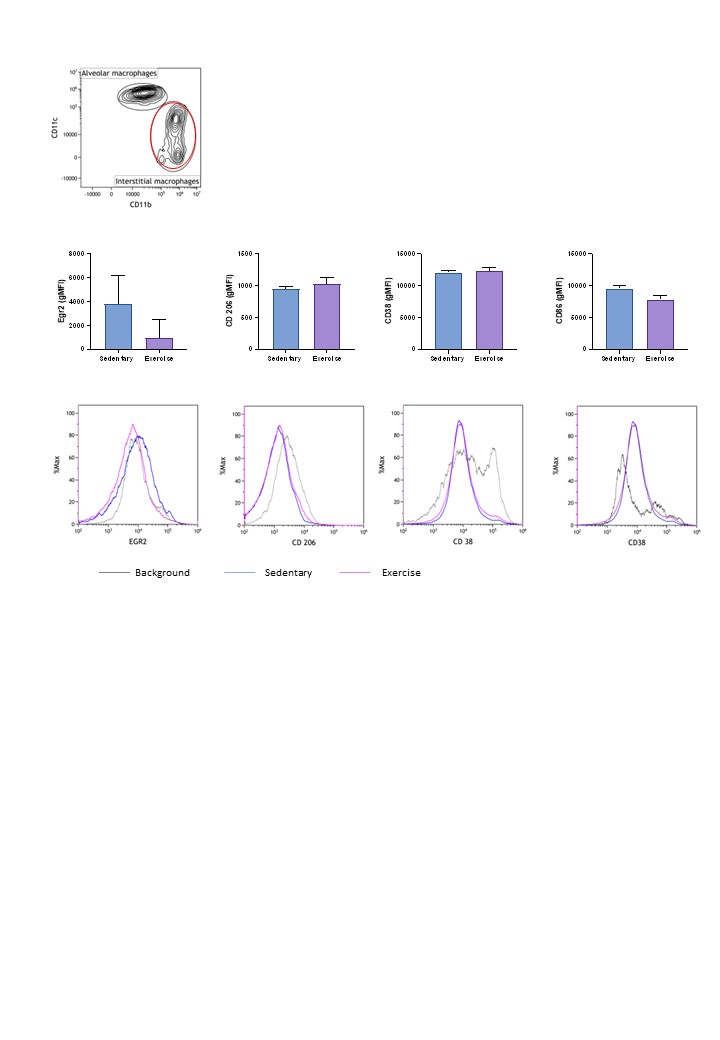


# Figure S6: Numbers of Neutrophils, Ams, IMs, CD4+ T cells, CD8+ T cells, B cells and NK cells at baseline and 12, 24, 48 hours after sepsis induction


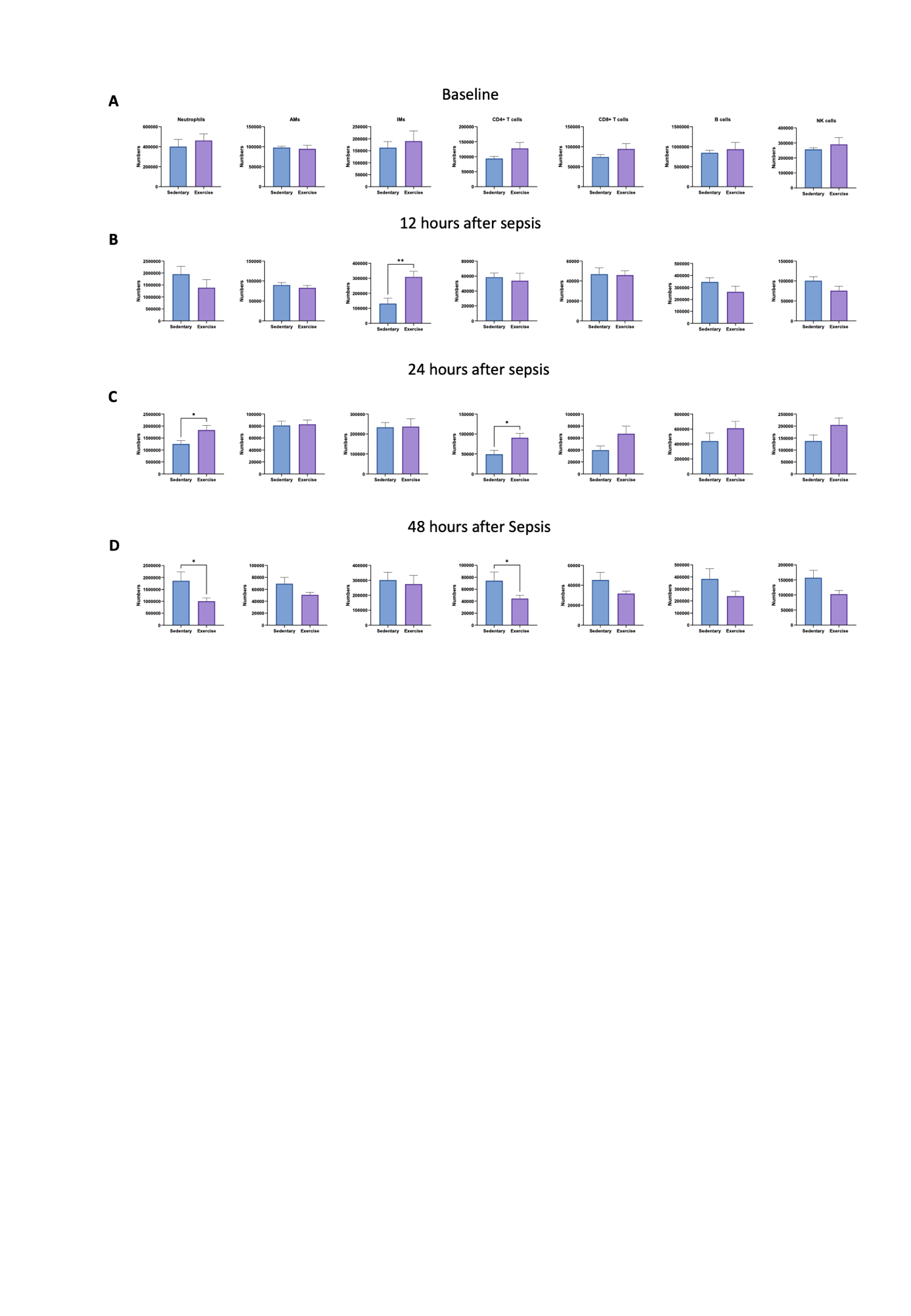


# Table S1: M-CASS scoring system

| Clinical criteria |  |  |  |  |
| --- | --- | --- | --- | --- |
| Fur aspect | Actively grooming | Dulling of hair coat | Rough hair coat | Piloerection |
| Activity | Normal activity | Reduced activity disturbed | No activity disturbed, reduced activity stimulated | Nil activity disturbed or stimulated |
| Posture | Normal | Slightly hunched, moving freely | Hunched with stiff movement/posture | Hunched with no movement stimulated |
| Behavior | Normal | Slow normal when disturbed | Abnormal disturbed, relocates only when stimulated | Abnormal when disturbed or stimulated, no relocation |
| Chest movements | Normal | Mildly dyspneic | Moderately dyspneic | Severely dyspneic with thoracic abdominal respiration |
| Chest sounds | No | Occasionally chirping | Frequently chirping | Wet chirping increased when stimulated |
| Eye lids | Normally opened spontaneously | Normally opened disturbed | Near closed when stimulated and disturbed | Closed disturbed, near closed stimulated |
| Body weight loss | 0%-5% | 5%-10% | 15%-20% | >20% |
| Score | 1 | 2 | 3 | 4 |
| Monitoring frequency | 12 hourly | 6 hourly during daytime | 4 hourly during daytime | Once identified as score 4, hourly checks at end of day, assess for end point |

# Table S2: Histologic severity score

Histologic severity score including scoring, localization and cell infiltrate

| Cell infiltration | Nothing | mild, inflammatory cells sporadically present in parenchyma; | moderate, single inflammatory lesion accounting for up to 10% lung parenchyma, | severe, multiple inflammatory lesions or pneumonia sites accounting for 10–50% lung parenchyma | complete, multiple inflammatory lesions and diffused consolidation or pneumonia accounting for >50% lung parenchyma |
| --- | --- | --- | --- | --- | --- |
| congestion | 0 | 0-10% | 10%-50% | 50%-80% | >80% |
| Score | 0 | 1 | 2 | 3 | 4 |

| Right lung | Cranial pole | Caudal pole | Middle pole |
| --- | --- | --- | --- |
| Left lung | Cranial pole | Caudal pole |  |

| alveolar cell infiltration | peribronchial | alveolar |
| --- | --- | --- |
| polynuclear cell infiltration | peribronchial | alveolar |

# Table S3: Number per experiments and reasons for excluded values

| **Experiment** | **N** | **Excluded values** | **Reasons** |
| --- | --- | --- | --- |
| Survival study | 24 | 1 | Failure of Anesthesia |
| Bacterial Load | 18 | 0 | Not Applicable |
| Flow cytometry | 72 | 6  7 | - Failure of staining  - Euthanasia before sampling |
| In vitro phagocytosis assay | 12 | 1 | Failure of bacterial administration |
| In vivo phagocytosis assay | 12 | 1 | Failure of bacterial administration |
| Lung cytokines measurement | 12 | 0 | Not Applicable |
| Plasma cytokines measurement | 12 | 0 | Not Applicable |
